# Direct Amperometric Sensing of Fish Nodavirus RNA Using Gold Nanoparticle/DNA-Based Bioconjugates

**DOI:** 10.3390/pathogens10080932

**Published:** 2021-07-23

**Authors:** Nadia Chérif, Mohamed Zouari, Fatma Amdouni, Marwa Mefteh, Ayoub Ksouri, Balkiss Bouhaouala-Zahar, Noureddine Raouafi

**Affiliations:** 1 Laboratory of Aquaculture (LR 16INSTM03), National Institute of Marine Sciences and Technologies, 28 Rue de 2 Mars, Salamboo 1934, Tunisia; fatmaamdouni12@yahoo.fr; 2 Laboratory of Analytical Chemistry and Electrochemistry (LR99ES15), Sensors and Biosensors Group, Tunis Faculty of Science, University of Tunis El Manar, Tunis 2092, Tunisia; zouariimohamed@gmail.com (M.Z.); marwaneftah1@gmail.com (M.M.); noureddine.raouafi@fst.utm.tn (N.R.); 3 Laboratory of Venoms and Therapeutic Molecules (LR16IPT08), 1” Place Pasteur, BP74, Pasteur Institute of Tunis, University of Tunis El Manar, Tunis 1002, Tunisia; ayoub.ksouri@pasteur.utm.tn (A.K.); balkiss.bouhaouala@fmt.utm.tn (B.B.-Z.); 4 Medical School of Tunis, University of Tunis El Manar, La Rabta, Tunis 1007, Tunisia

**Keywords:** betanodavirus, amperometry, nanosensors, RT-PCR, fish, diagnosis

## Abstract

We describe the design of a simple and highly sensitive electrochemical bioanalytical method enabling the direct detection of a conserved RNA region within the capsid protein gene of a fish nodavirus, making use of nanostructured disposable electrodes. To achieve this goal, we select a conserved region within the nodavirus RNA2 segment to design a DNA probe that is tethered to the surface of nanostructured disposable screen-printed electrodes. In a proof-of-principle test, a synthetic RNA sequence is detected based on competitive hybridization between two oligonucleotides (biotinylated reporter DNA and target RNA) complimentary to a thiolated DNA capture probe. The method is further validated using extracted RNA samples obtained from healthy carrier *Sparus aurata* and clinically infected *Dicentrarchus labrax* fish specimens. In parallel, the sensitivity of the newly described biosensor is compared with a new real-time RT-PCR protocol. The current differences measured in the negative control and in presence of each concentration of target RNA are used to determine the dynamic range of the assay. We obtain a linear response (R^2^ = 0.995) over a range of RNA concentrations from 0.1 to 25 pM with a detection limit of 20 fM. The results are in good agreement with the results found by the RT-qPCR. This method provides a promising approach toward a more effective diagnosis and risk assessment of viral diseases in aquaculture.

## 1. Introduction

Among the most reported viral infections in marine aquaculture, viral encephalopathy and retinopathy (VER—also known as viral nervous necrosis, VNN) caused by betanodaviruses (piscine nodavirus) is one of the most highly impactful pathogens in hatcheries and grow-out fish facilities. Betanodaviruses are found in wild and cultured fish across a broad geographical range, having been isolated from and/or associated with outbreaks of VER in almost all continents [1]. Piscine nodaviruses are recognized for their lack of host specificity [2] and are characterized by a genome of positive-sense, single-stranded RNA. Belonging to the genus *Betanodavirus* and family *Nodaviridae* [3], the genome of the viral nervous necrosis virus (VNNV) consists of two segments named RNA1 (3.1 Kb) and RNA2 (1.4 Kb), coding for the RNA-dependent RNA polymerase and the coat protein, respectively. Based on phylogenetic analysis of the RNA2 segments (T4 variable region), betanodavirus isolates have been classified into four genotypes, currently accepted by the International Committee on Taxonomy of Viruses (ICTV): striped jack nervous necrosis virus (SJNNV), tiger puffer nervous necrosis virus (TPNNV), barfin flounder nervous necrosis virus (BFNNV) and redspotted grouper nervous necrosis virus (RGNNV) [3,4]. Moreover, reassortant isolates harboring RGNNV and SJNNV genomic segments (in both RNA1/RNA2 combinations: SJNNV/RGNNV and RGNNV/SJNNV) have been reported [5,6]. Until 2019, only outbreaks in sea bass species had been registered in the Tunisian and North African fishing industry, caused by the redspotted grouper virus RGNNV genotype [7,8,9]; however, new RGNNV/SJNNV reassortants have been recently introduced and are causing severe economic loss in sea bream species, previously considered a healthy carrier species (Chérif et al., personal communication).

Multiple nodavirus diagnostic methods have been reported testing blood, gills and caudal fin samples [10,11,12,13,14,15,16,17,18,19,20,21,22,23]. In recent years, RT-PCR detection has given way to RT-qPCR assays targeting both genomic segments [11,14,19,20]. In addition, a real-time procedure combined with high-resolution melting (HRM) has been proposed for NNV detection and genotyping [18]. Other methodologies for rapid and accurate nodavirus detection have recently been developed, including: loop-mediated-isothermal-amplification (LAMP) [24], real-time LAMP assays [25], and an immunomagnetic reduction (IMR) assay, which employs magnetic nanoparticles coated with dextran and antibodies [26]. Despite significant progress in NNV detection, recent advances linked to the use of nano sensing, already reported for human and animal viruses [27,28,29,30,31,32,33,34,35], can provide a substantial improvement in fish virus diagnostics. Herein, we demonstrate an electrochemical biosensor to further facilitate betanodavirus detection based on modified nanostructured disposable electrodes harboring a thiolated DNA probe complementary to a conserved region of the nodavirus RNA2 segment.

## 2. Material and Methods

### 2.1. Primer and Probe Design

**Figure 1:**
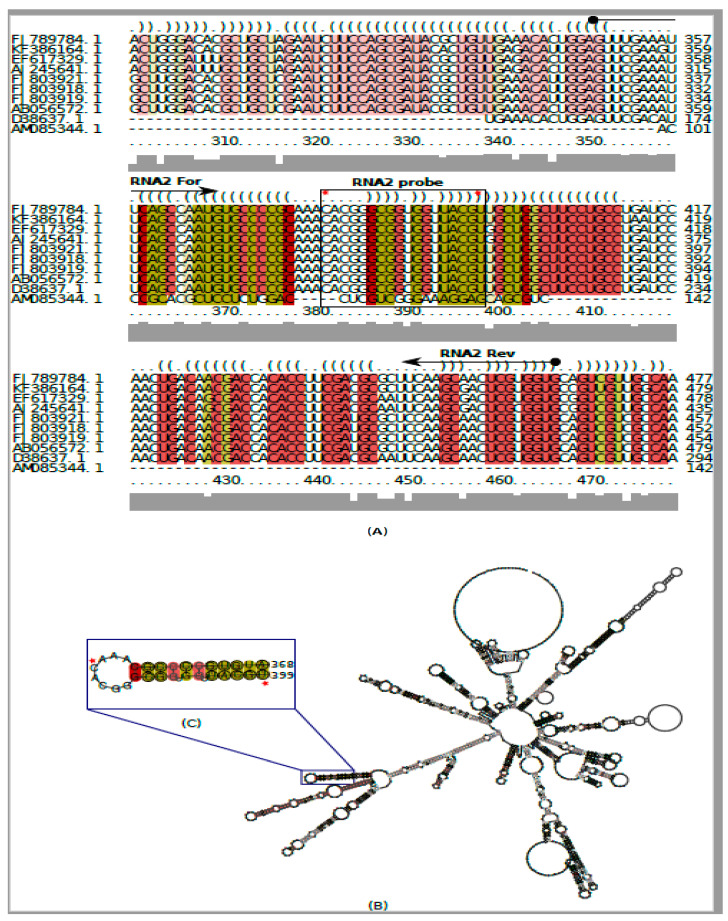
(**A**): Multiple sequence alignment of betanodavirus RNA2 nucleic sequences. GenBank accession numbers are listed. RNA2 probe hybridizes to positions 381–399 of the RGNNV RNA2 sequence (GenBank accession number: FJ789784). (**B**): 2D/3D structure of RNA2 generated by RIBOSUM-like similarity scoring. (**C**): Secondary structure of the RNA as end of branch with a very small, conserved hairpin.

Primers and probe were designed using the “Primer Express Software v2.0” (Applied Biosystems, Foster City, CA, USA) following standard procedures targeting a highly conserved region of the piscine nodavirus genome, based on multiple sequence alignment, including representative sequences that are publicly available in GenBank (Figure 1A) (http://www.ncbi.nlm.nih.gov (accessed on 5 March 2021)). Multiple alignment of RNA sequences (MSA) was performed using LocARNA software. The MSA algorithm in the present case is mainly based on RIBOSUM-like similarity scoring and the realistic gap cost. The MSA output was adopted as a matrix to visualize the consensus and pattern sequences [36]. In addition, RNA2 segment encoding for the capsid protein was subjected to comparative structural analysis using Freiburg RNA Tools (LocARNA—Alignment & Folding) in order to identify structural similarities and divergences of the nodavirus sequences. RIBOSUM-like similarity scoring was adopted as a feature of the generated alignment and the 2D/3D RNA structure including hairpins and loops was extensively characterized [37].

For the RT-qPCR protocol, new oligonucleotides were designed to target and amplify a conserved 116-bp-long region of the viral genome, localized in the RNA2 segment and encoding for the capsid protein (Figure 1A). The primers and probe sequences were as follows: RNA2 FOR 5′- GTTCGAAG TTCAGCCAATG-3′, RNA2 REV 5′-TTCAAGCGACTCGTGGTG-3′ and RNA2 probe 5′-6FAM-CACGGGCGGTGGTTACGT-BHQ1-3′ (Figure 1B). Oligonucleotides designed to evaluate AuNPs/SPCEs analytical performance and its application in nodavirus RNA2 detection were as follows: SH-capture DNA probe 5′-GTTGGATCAGGCAGGAAG-3′, biotinylated DNA 5′-CAACCTAGTCCGTCCTTC-3′ and target RNA 5′-CAACCUAGUCCGUCCUUC-3′.

### 2.2. Assay Principle

**Figure 2:**
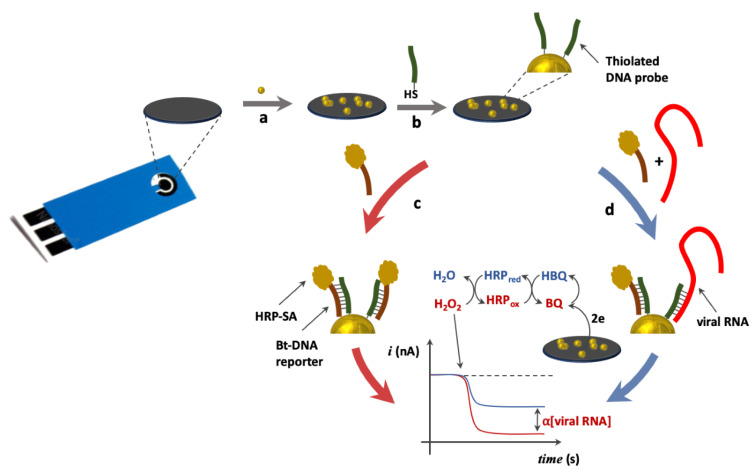
Schematic illustration of the competition-based method for the detection of the viral RNA. Steps: (**a**) gold electrochemical deposition, (**b**) bioconjugation with the thiolated DNA capture probe, (**c**) addition of the biotinylated reporter probe and (**d**) addition of the sample containing the viral RNA and the thiolated reporter probe. A higher current is observed in case (**c**), where all the capture probes are solely hybridized with the biotinylated reporter probe, while in case (**d**) the competition results in a lower current. The current difference is proportional to the RNA_target_ concentration.

The step-by-step strategy of the direct competitive assay developed for nodavirus RNA detection was based on the use of SH-DNA/AuNPs/SPCEs, as schematically illustrated in Figure 2. Precisely, a competitive hybridization occurred between two strands of oligonucleotides (biotinylated DNA and target RNA) facing the same thiolated DNA probe (SH-DNA), complementary to a conserved region of the nodavirus RNA2 (steps a and b). First, the thiolated DNA probe was immobilized on the surface of the electrode via an Au-S bond established between the thiol group of the HS-DNA probe (0.05 μM) and the gold nanoparticles (AuNPs) present on the surface of the electrode. Then, the immobilized DNA probe was hybridized with a biotinylated fragment (Bt-DNA reporter). Following the hybridization process, a streptavidin protein conjugated to horseradish peroxidase (HRP) (Strep-HRP) with a high affinity to biotin was incubated. By this method, it is possible to differentially generate a current from the reduction reaction of the benzoquinone produced by the oxidation of hydroquinone (HQ) and the reduction of hydrogen peroxide (H_2_O_2_) catalyzed by HRP. When the probe is only hybridized to biotinylated DNA, the current produced is at its maximum (step c). Then, after adding our synthetic target RNA or viral RNA in competition, the amperometric response registered a decrease proportional to the remaining immobilized biotinylated DNA fragments (step d). All of the required pre-treatment to avoid contamination adsorption was applied.

### 2.3. Preparation of the Gold Nanoparticles-Modified Screen-Printed Electrodes

#### 2.3.1. Apparatus and Electrodes

Amperometric measurements were carried out using an EmStat3 Blue potentiostat (PalmSens, The Netherlands). Experiments were designed and data were controlled by PSTrace version 4.4. All measurements were carried out at room temperature (RT). The transducers employed were gold nanoparticle-modified screen-printed carbon electrodes (AuNPs-SPCEs) (DRP-110GNP, DropSens), formed by a 4-mm-diameter carbon working electrode and printed carbon and silver wires as counter and pseudo-reference electrodes, respectively. A specific cable connector (DRP-CAC, DropSens) interfaced between the AuNPs/SPCEs and the EmStat3 potentiostat. A Raypa steam sterilizer, biological fumehood (Telstar Biostar) and a temperature freezer (New Brunswick Scientific) were also employed.

#### 2.3.2. Immobilization of DNA Probe

In the first step, the DNA capture probe was immobilized onto the electrodes as previously described by Zouari et al. [38,39]. Briefly, the protocol consists of casting a volume of 10 µL of a 0.05 µM HS-DNA capture probe solution (prepared in Tris-EDTA, pH 8.0) onto the working electrodes, incubated overnight at 4 °C. After washing, the modified electrodes were blocked with 10 µL of 0.1 mM mercaptohexanol aqueous solution (in Tris-EDTA, pH 8.0) for 5 min and 1% (*w*/*v*) BSA (in Tris-EDTA, pH 8) for 60 min, to minimize the non-specific adsorption of the enzyme marker.

#### 2.3.3. Competitive Hybridization

After depositing the SH-DNA capture probe, the second step consists of depositing onto the modified electrodes 10 μL of a mix containing a fixed concentration of biotinylated DNA and different concentrations of the synthetic target RNA (RNA_target_) ranging from 0.1 to 25 pM (in PBS, pH 7.5) and incubating them at RT for 45 min to hybridize. Then, the electrodes were washed with deionized water, dried by gentle nitrogen flux. In a second step, 10 µL of a 0.05 U·mL^−1^ Strep-HRP solution, which was prepared in a commercial blocker case in solution, was added and incubated for 30 min at RT. Last, the SH-DNA/AuNPs/SPCEs were washed with deionized water and dried by gentle nitrogen flux.

#### 2.3.4. Amperometric Detection

The modified electrodes were immersed in an electrochemical cell containing 10 mL of 0.05 M of phosphate buffer (pH 6.0) and freshly prepared 1.0 mM HQ. The amperometric measurements were performed while keeping the agitation of the solution in which the electrodes were immersed and while imposing a detection potential of −0.20 V vs Ag/AgCl. As soon as the current generated began to stabilize, 50 μL of a 0.1 M solution of hydrogen peroxide was added. The current difference (Δi) was related to that measured in the non-template control (NTC) and the one with RNA_target_ (synthetic standards). Data corresponded to the average of the three replicates and the confidence intervals were calculated for α = 0.05. The detection limit (LOD) was determined from the current difference (Δi) using the synthetic target RNA three times, and the standard deviation of the current values was determined from ten independent amperometric measurements.

### 2.4. Fish Samples, RNA Extraction and Biosensing Assay Optimization

Two sets of brain tissue samples from *Dicentrarchus labrax* and *Sparus auarata* fish specimens were used as targets for the RT-qPCR and AuNPs/SPCEs optimization studies. Fish samples were selected from the National Institute for Marine Sciences and Technologies (INSTM) tissue banks that were previously characterized for the presence of betanodavirus [40], namely SS and AS, related to the presence or absence of VNN clinical signs, respectively (Table 1). Biosensing assays were performed on pools of 10 brains, which were homogenized and clarified at 3000× *g* for 15 min at 4 °C in a refrigerated centrifuge. Aliquots of the viral suspensions were applied to cultures of SSN-1 cells [41]. Viral titer, expressed as TCID_50_/mL, was determined according to the Spearman-Karber method [42]. Total RNA was extracted from 200 μL of fish tissue homogenates using the PureLink™ Micro-to-Midi Total RNA Purification System (Invitrogen) according to the manufacturer’s instructions. Measurements of the absorbance at 260 nm with a Nanodrop 1000 spectrophotometer (Thermo Fisher Scientific, Delaware city, USA) confirmed that the isolated RNA was pure, while also extrapolating its concentration. AS and SS fish RNA were hybridized in direct competition with the synthetic target RNA in triplicates. The difference in measured currents (Δi) corresponded to the difference between the current measured in the non-template control (NTC) and the current measured with RNA targets.

### 2.5. Optimization of the RT-qPCR Analytical Assay

A TaqMan RT-qPCR test was optimized in order to compare the detection specificity and sensitivity of the biosensor. The reaction started with a reverse transcription step (50 °C for 5 min), the activation of the polymerase at 95 °C for 20 s and 40 cycles of cDNA amplification (95 °C for 30 s, 55 °C for 30 s and 72 °C for 30 s). It was carried out in a 7500 Real Time PCR system using the TaqMan^®^ Fast virus One-Step kit (Applied Biosystems). The sensitivity of the reaction has been evaluated for the detection of the RGNNV genotype in terms of the minimal viral titer calculated (TCID_50_/mL). RNA was extracted from 200 μL of supernatant from a previously titrated viral stock (10^7^ TCID_50_/mL), and ten-fold serial dilutions were used to test real-time RT-qPCR sensitivity. The intra-assay variation was determined in a real-time PCR run analyzing the standard deviation (SD) obtained from the mean of three replicates. The sensitivity and efficiency of the real-time RT-qPCR were assessed by creating a standard curve. The specificity of the method was assessed by testing the newly designed set of primers and probing low virus titer asymptomatic samples, in addition to other available viruses: viral hemorrhagic septicemia virus genotype I (VHSV-I) and lymphocystis disease virus (LDV) genotype VII.

## 3. Results

### 3.1. 2D/3D RNA Structure

The MSA of 10 distinct nodavirus sequences, including the sequence of the local strain of RGNNV (GenBank accession number: FJ789784), showed highly conserved regions and indicated that fish nodavirus RNAs shared a remarkable conserved consensus sequence (Figure 1A). The 2D/3D conformational structure, based on RIBOSUM-like similarity scoring and realistic gap cost, exhibited several hairpins of different nucleotide sizes separated by small and mid-size loops, within the RNA structure (Figure 1B). The most conserved primary sequence and open 2D/3D structure were used to design the RNA2 forward and RNA2 reverse primers. Similarly, the RNA2 5′-6FAM/BHQ1-3′ probe from positions 381 to 399, with a very high nucleotide similarity percentage, was designed. This fragment was present in the secondary structure of the RNA2 as the end of a branch with a very small, conserved hairpin (Figure 1C).

### 3.2. Analytical Performance of AuNPs/SPCEs

#### 3.2.1. Optimization of Parameters Involved in the Signal Amplification

Different variables such as DNA probe concentration, hybridization time with the target RNA, and AuNPs/SPCEs concentration, which may affect the analytical performances of the designed bioelectrode, were optimized. In addition, other parameters such as the pH value, the detection potential and the concentrations of H_2_O_2_ and hydroquinone were investigated. In particular, the cyclic voltammetry curves recorded in the absence and the presence of different AuNPs/SPCEs concentrations were compared. In addition, data obtained showed that the biosensor response was SH-DNA probe concentration-dependent and with respect to the different concentrations tested (1 µM, 0.5 µM, 0.1 µM, 0.05 µM and 0.01 µM), 0.05 μM of ssDNA was considered enough to stabilize the AuNPs (Supplementary Material Figure S1).

#### 3.2.2. Dynamic Range and Detection Limit

**Figure 3:**
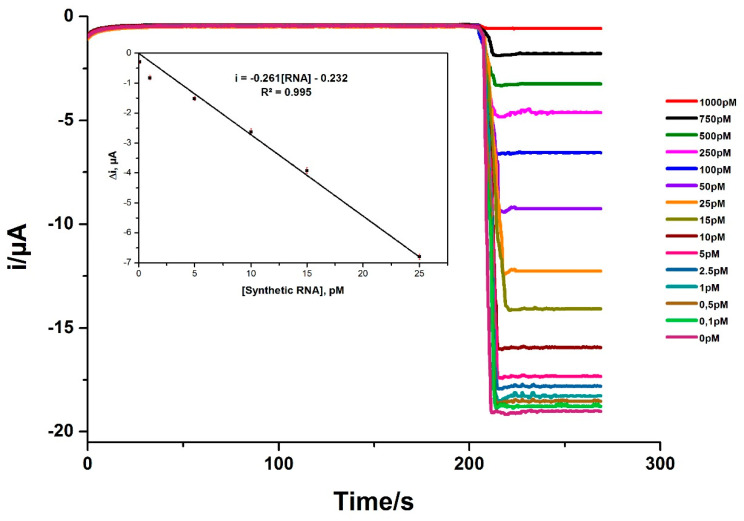
Amperometric measurements with synthetic RNA target at different concentrations. Inset: Linear portion of the curve between 100 fM and 25 pM.

Figure 3 shows the amperometric response measured under optimal conditions for different concentrations of synthetic RNA. As expected, and in accordance with the concept of competitive hybridization, we found that at the highest concentrations of the target RNA, the highest amperometric signal decreased. The current differences obtained in the absence and in the presence of each concentration of target RNA were used to determine the dynamic range of the assay. We obtained a linear response (R^2^ = 0.995) over a range of RNA concentrations from 0.1 to 25 pM. Furthermore, a limit of detection (LOD) of 20 fM for the synthetic target RNA was estimated from the standard deviation of the current values obtained for ten independent electrochemical measurements.

#### 3.2.3. Nodavirus Detection on Field Samples Using AuNPs/SPCEs

**Figure 4:**
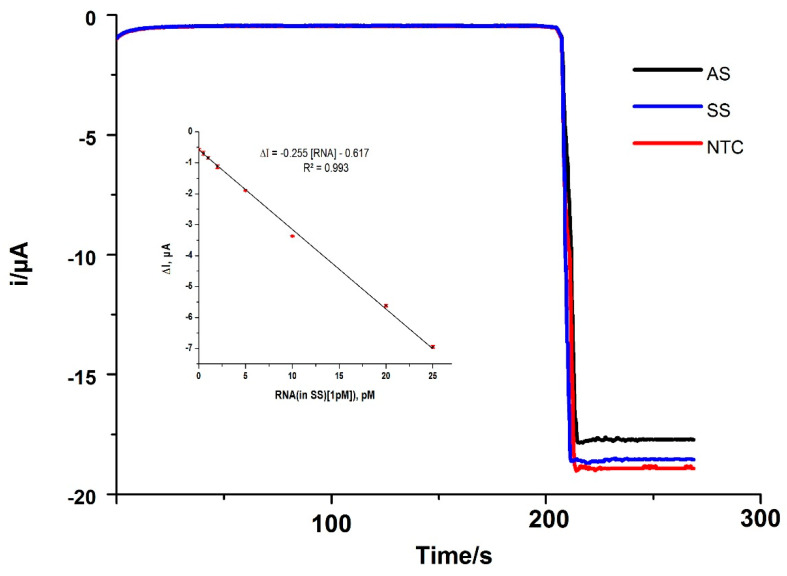
Amperometric responses measured with the nodavirus-developed biosensors for 100 ng raw RNA_target_ extracted from two real samples (AS and SS) as compared to the negative control with no target (NTC). Inset: Linear portion of the curve by standard addition method to the real sample.

The potential of the nano-bioconjugate for the detection of betanodavirus in a real scenario was further investigated. The bioelectrode was assembled by the same procedure and instead of the synthetic RNA, 10 μL of the RNA-containing fish tissue sample was used. Both infected (SS) and healthy carrier (AS) field samples were analyzed. Interestingly, no matrix effect was apparent when using amounts lower than or equal to 100 ng of extracted RNA from field samples. Indeed, the slope of the linear calibration curve was plotted using field sample (SS) RNA as a target to be equal to 0.255 ± 0.03 µA·pM^–1^ and was not significantly different from the slope obtained with standard synthetic RNA solutions (0.261 ± 0.07 µA·pM^–1^) (t_exp_ = 3.098 < t_tab_ = 4.303; n = 3, α = 0.05). Consequently, the quantification of synthetic RNA could be simply accomplished by extrapolation of the current values measured for the RNA_target_ extracted from the calibration curve obtained with standard solutions of RNA. The comparison of amperometric signals (Δi) of nodavirus infected specimens with the negative control determined whether the samples were positive for nodavirus RNA or not. More interestingly, the samples from infected specimens exhibited differential current responses with respect to the NTC, which can be indirectly related to the RNA loading in each sample. As expected, nodavirus copy number determination showed (1.04 ± 0.50) × 10^8^ and (5.88 ± 0.70) × 10^5^ copies per µL of RNA_target_, corresponding to clinically infected (SS) and healthy carrier (AS) samples, respectively (Figure 4). No copy number was calculated for the NTC.

### 3.3. RT-qPCR Assay Analytical Sensitivity, Specificity and Fish Analysis

**Figure 5:**
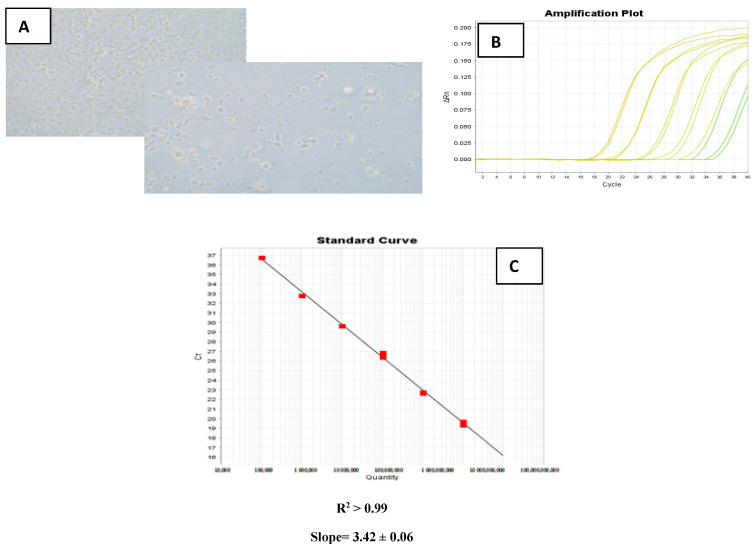
(**A**): Nodavirus isolation and characterization on SNN-1 cell line showing strong CPE seven days post-inoculation with a nodavirus positive sample incubated at 25 °C. (**B**): Analytical sensitivity of the real-time TaqMan PCR: Amplification curves obtained by testing tenfold serial dilutions of quantitated RNA standards. The *x*-axis shows the number of PCR cycles. The *y*-axis shows the fluorescence values on a logarithmic scale. (**C**): Standard curve generated from the above data. The *x*-axis indicates the logarithm of the RNA concentration expressed in copy number. The *y*-axis indicates the Ct value.

RT-qPCR efficiencies were calculated from the slopes given by the Applied Biosystems instrument software. The quantitative real-time PCR efficiency (E) of one cycle in the exponential phase was calculated according to the equation: E% = (10^−1/S^ − 1) × 100, where S is the slope of the linear fit. The regression analysis yielded a correlation coefficient R^2^ > 0.99. A slope value of 3.42 ± 0.06 indicated an amplification efficiency of the CP gene of 96% (Figure 5).

No amplification was detected in nodavirus negative controls or the no-template controls up to 40 cycles. No Ct values were obtained when testing outgroup viral RNAs such as VHSV and LDV (data not shown).

The diagnostic effectiveness of the RT-qPCR assay was validated to detect the nodavirus CP gene isolated from diseased and healthy carrier fish (Table 1). Viral RNA loads showed different copy numbers as expected. The lowest concentration (3.57 ± 0.019 × 10^6^) was obtained from an asymptomatic sea bream sample (AS). The analysis of clinically infected sea bass specimens (SS) yielded RNA charges of 2.52 ± 0.023 × 10^10^.

## 4. Discussion

Early-stage detection of diseases is one of the biggest challenges in aquaculture. Recent work suggests that DNA-based nano-biosensors could be used to provide simple, fast, cost-effective, sensitive and specific detection of some genetic conditions, cancer and various infectious diseases [43]. Here, we investigated the conformational structure of the nodavirus RNA structure. Based on the optimal primary sequence and RGNNV RNA2 hairpins and loops, we designed primers and a probe to perform proof-of-principle assays for two novel rapid, sensitive and specific tools for early VER diagnosis. Specifically, a novel gold nanoparticle-based amperometric biosensor for direct nodavirus detection, based on the competitive hybridization of nucleic acids and a new RT-qPCR technique. Data demonstrate the high potential of the nanometric gold bioconjugates for the ultrasensitive detection of target RNA at extremely low concentrations, down to the fM level. To the best of our knowledge, this is the first AuNP-DNA nano–gold bioconjugate utilized to sensitively detect betanodavirus genetic material, with no need for a PCR amplification.

The assembled biosensor was used to detect a fish nodavirus in *D. labrax* and *S. aurata* samples using optimized reaction conditions, and the nodavirus copy number determinations showed 1.04 ± 0.50 × 10^8^ and 5.88 ± 0.70 × 10^5^ copies/µL of RNA_target_, corresponding to symptomatic (SS) and healthy carrier (AS) specimens, respectively. We compared these results with viral RNA loads obtained using the new RT-qPCR protocol, which provided slightly greater sensitivity. The lowest positive RNA concentration by RT-qPCR, 3.57 ± 0.019 × 10^6^, was obtained from an asymptomatic (AS) sea bream sample, whereas the analysis of clinically infected (SS) sea bass specimens yielded RNA charges up to 2.52 ± 0.023 × 10^10^.

The use of a nanoparticle-based lateral flow biosensor (LFB) for the detection of NNV amplification products enabled accurate and fast diagnostics under field conditions, although it did not increase sensitivity compared to RT-qPCR assays [22,44]. Other LFB biosensors exist for shrimp virus detection [22,45,46,47] as well as for fish viruses including infectious spleen and kidney necrosis virus [48] and cyprinid herpesvirus 3 or Koi herpesvirus [49]. A comparison of the analytical performance of the nodavirus AuNPs/SPCEs biosensor established here with that of the LFB reported by [44] demonstrated a higher sensitivity compared to the LFB, which was able to detect 135 pg of nodavirus RNA in biological samples.

Current control strategies for fish viruses have not changed substantially from those included in previous reviews [50,51], namely, good husbandry practices, including biosecurity and sanitation. The new AuNPs/SPCE biosensor offers great potential for commercial kit development and use in field assays by fish farmers. The assay will allow for disease monitoring without time-consuming and costly procedures. The prevention of vertical transmission through the selection of NNV-free broodstock using such innovative tools will be of great utility, as well. Indeed, the assay offers outstanding properties for signal amplification, improved sensitivity and lower LOD. However, the challenge will be to develop a nanoplatform that fulfills the requirements of ease of fabrication, storage stability and high specificity while minimizing time and ability to detect very low levels of target concentrations at low cost. Fortunately, all of the reagents and disposable electrodes used are commercially available, making this biosensor particularly attractive for use by nonspecialized personnel for routine determinations.

## Figures and Tables

**Table 1:** Description of results obtained from RT-qPCR and AuNPs/SPCEs betanodavirus quantification. Ct mean values and RNA copy number of the coat protein (CP) gene related to the fish status and the sample type are listed.

Sample ID	Fish Species	Clinical Signs	Matrix	CPE	RT-qPCR Results		AuNPs/SPCEs Results
					Ct	Copy Number	Copy Number
AS	Sea bream	NO	Brain homogenates	-	33.71 ± 0.107	3.57 ± 0.019 × 10^6^	5.88 ± 0.7 × 10^5^
SS	Sea bass	Yes	Brain homogenates	+	21.78 ± 0.028	2.52 ± 0.023 × 10^10^	1.04 ± 0.5 × 10^8^

## Data Availability

The data that support the findings of this study are available on request from the corresponding author. Some of the data are not publicly available due to privacy or ethical restrictions.

## Supplementary Materials

The following are available online at https://www.mdpi.com/article/10.3390/pathogens10080932/s1, Figure S1: Optimization of parameters involved in the signal amplification. Optimization of (A) chronoamperometry curves for the gold nanoparticles electrodeposition at different concentrations, (B) the incubation time for AuNPs/SH DNA bioconjugates and (C) optimization of the SH-DNA probe concentrations.
