# Supplementary material for: Direct Amperometric Sensing of Fish Nodavirus RNA Using Gold Nanoparticle/DNA-Based Bioconjugates
Source: Pathogens. 2021 Jul 23;10(8):932. doi: 10.3390/pathogens10080932 (PMC8398327; doi:10.3390/pathogens10080932)
Supplement: Supplementary file 1 [file pathogens-10-00932-s001.zip › pathogens-1175308-supplementary.pdf]

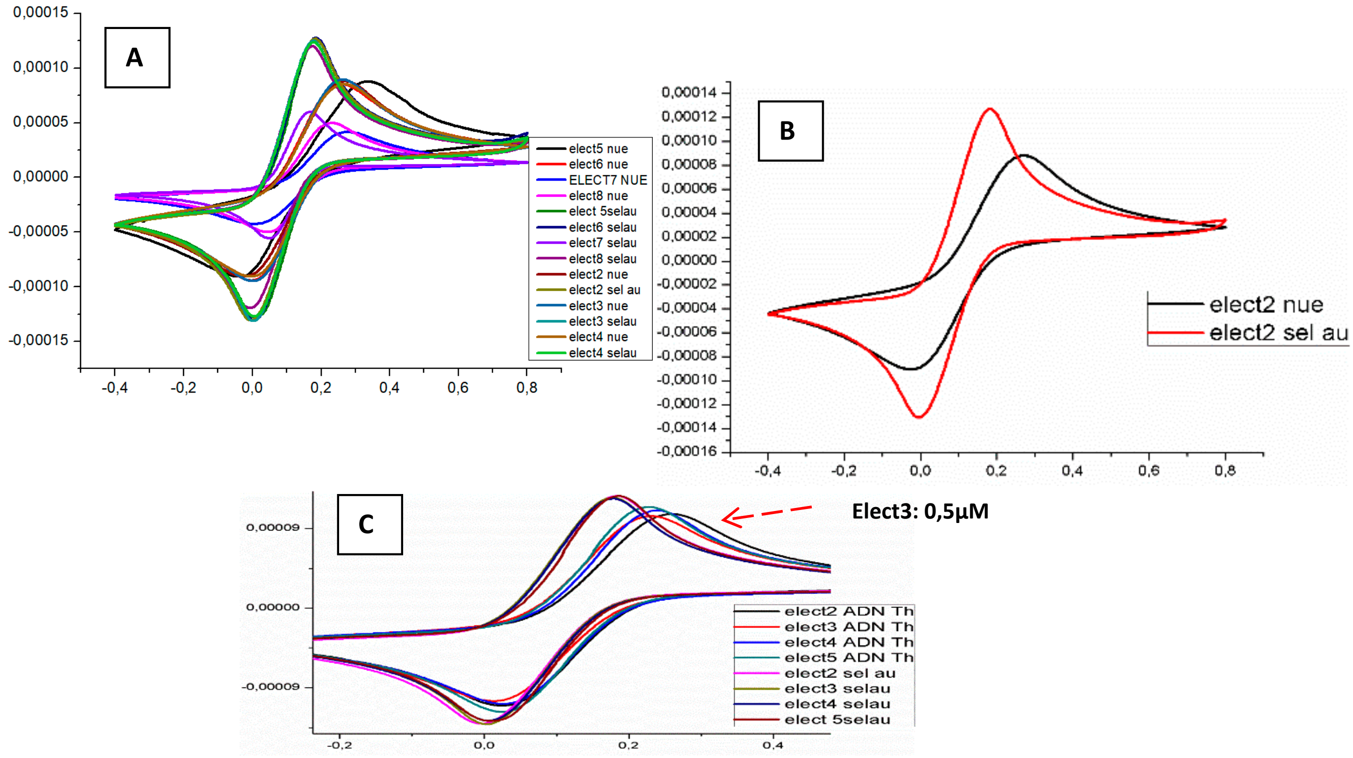

Figure 1. Optimization of parameters involved in the signal amplification. Optimization of (A) chronoamperometry curves for the gold nanoparticles electrodeposition at different concentrations, (B) the incubation time for AuNPs/SH DNA bioconjugates and (C) optimization of the SH-DNA probe concentrations.
